# Inferring the Dysconnection Syndrome in Schizophrenia: Interpretational Considerations on Methods for the Network Analyses of fMRI Data

**DOI:** 10.3389/fpsyt.2016.00132

**Published:** 2016-08-03

**Authors:** Brian H. Silverstein, Steven L. Bressler, Vaibhav A. Diwadkar

**Affiliations:** ^1^Department of Psychiatry and Behavioral Neurosciences, Brain Imaging Research Division, Wayne State University, Detroit, MI, USA; ^2^Center for Complex Systems and Brain Sciences, Florida Atlantic University, Boca Raton, FL, USA

**Keywords:** brain networks, fMRI methods, schizophrenia, connectivity analysis, dysconnection syndrome

## Abstract

Schizophrenia has long been considered one of the most intractable psychiatric conditions. Its etiology is likely polygenic, and its symptoms are hypothesized to result from complex aberrations in network-level neuronal activity. While easily identifiable by psychiatrists based on clear behavioral signs, the biological substrate of the disease remains poorly understood. Here, we discuss current trends and key concepts in the theoretical framework surrounding schizophrenia and critically discuss network approaches applied to neuroimaging data that can illuminate the correlates of the illness. We first consider a theoretical framework encompassing basic principles of brain function ranging from neural units toward perspectives of network function. Next, we outline the strengths and limitations of several fMRI-based analytic methodologies for assessing *in vivo* brain network function, including undirected and directed functional connectivity and effective connectivity. The underlying assumptions of each approach for modeling fMRI data are treated in some quantitative detail, allowing for assessment of the utility of each for generating inferences about brain networks relevant to schizophrenia. fMRI and the analyses of fMRI signals provide a limited, yet vibrant platform from which to test specific hypotheses about brain network dysfunction in schizophrenia. Carefully considered and applied connectivity measures have the power to illuminate loss or change of function at the network level, thus providing insight into the underlying neurobiology which gives rise to the emergent symptoms seen in the altered cognition and behavior of schizophrenia patients.

## Introduction

Schizophrenia is the consummate “epigenetic puzzle” (1). Psychiatrists, for the most part, know it when they see it in the clinic (2), yet its biological *origins* are utterly obscure, given that its etiology and genetic bases are poorly understood. We seem to *know* much but *understand* very little (3). Scientists have settled on the view that schizophrenia is a polygenic disorder (4, 5) where multiple genes (that themselves exert pleotropic effects) confer vulnerability, but wherein the frank symptoms of the disorder themselves emerge from a complex (and plausibly in-deterministic) genetic-development-environmental interplay (6–9).

Lack of understanding of causative pathways (in addition to other factors such as phenotypic heterogeneity) is a limiting constraint on efforts at prevention, early intervention, and/or treatment of schizophrenia (10). Yet, as is evident in other fields of medicine (most notably cardiology and oncology), successful medical treatment and management does not necessarily depend on identifying deterministic causal pathways toward disease. Rather, understanding of vulnerability measures is sufficient, as long as there is a clear characterization of the pathophysiologic *mechanisms* underlying the disease. A grasp of basic biological mechanisms drives the development of targeted therapies while simultaneously providing objective biomarkers that can inform treatment efficacy (11, 12). Psychiatry has long been criticized for focusing on emergent *effects* of disease, while having an insufficient focus on understanding *mechanisms* of disease. As a result, many emergent effects are insufficiently constrained by analytic approaches designed to explicitly characterize mechanisms. The argument is that these limitations essentially limit nosology and treatment (13). Here, we reiterate the widely held view that the mechanisms of diseases like schizophrenia will most profitably be understood by focusing on, to put it simply, how the brain is “not working.” More pertinently, we accept the position that the current state of acquiring and modeling brain signals suggests that brain–behavior relationships may best be understood from the position of *macroscopic* brain network interactions (14), a scale that may most proximately map cognitive and sensorimotor function to its underlying correlates. We are aligned with the position that these investigations are a matter of discovery, and subscribe to the distinction between “true” models of brain function (whatever those may be) and “likely” models of brain function (15), where the former are theoretical constructs, whereas the latter are empirically discoverable from neuroimaging data. We will assert that these ontological subtleties are of direct (and not merely academic) relevance to the study of schizophrenia: if understanding brain mechanisms subserving normal function is a process of discovery, by corollary, understanding these in the context of schizophrenia is also a process of discovery. Moreover, this process is constrained by quantitative models, and the assumptions therein, that are applied to any class of neuroimaging data. Thus, understanding disconnection in schizophrenia is an *inference*, and the class of models applied will provide constraints on the type of inference that can be drawn (16).

What follows is a compendium of previously advanced ideas motivating schizophrenia as a syndrome of functional disconnection, or more accurately, “dysconnection”: this is generally understood as the abnormal integration of signals across the brain (17). These ideas are not novel to this review and, in fact, have remained in place since the earliest conceptions of schizophrenia itself. However, over time the idea of dysconnection, once a general construct, has been systematically hewn to a point where it can be seen as having clear bases in translational neuroscience (18–20). We willfully restrict our scope to the approaches used in the analyses of functional magnetic resonance imaging (fMRI) signals. Thus, while much of what we review strictly extrapolates to fMRI studies in schizophrenia, many of the quantitative approaches that we review are not specific to fMRI time series analyses.

Much has been written about fMRI, its neurophysiological bases, the relative advantages of the signal, and its limitations for assessing brain function (21–24). Moreover, the comparative merits and demerits of different imaging techniques for discovering connection and disconnection in the brain is a viable topic but beyond our scope (25, 26). As a technique, fMRI is neither mercurial nor worthless; rather the technique provides access to credible signals that under appropriate analytic constraints can tell us something interesting about how the brain works.

## Organ Systems and Phenotypes

As understood from the early origins of psychiatric taxonomy, the body’s only organ system of direct relevance to schizophrenia (and indeed all psychiatric illnesses) is the brain, and the brain’s *functional* properties were seen as the most salient in this regard (27, 28). Psychiatric phenotypes have always fundamentally been defined by behavioral abnormalities (29). If the “mind–body” problem is narrowly defined as the problem of understanding how mental states emerge from the physical states of the body (30, 31), then understanding pathophysiological mechanisms underlying schizophrenia is a special case of the mind–body problem. That is, if “neural” processes drive normal behavior, then abnormal behavior (i.e., such as those observed in schizophrenia) must result from abnormal neural processes. A challenge then is to identify abnormal neural mechanisms that lie in a straightforward relationship with the phenotypic characteristics of schizophrenia itself. Given that the dialectic regarding the mind–body problem remains active, it is self-evident that understanding neural mechanisms associated with schizophrenia is a non-trivial problem.

The term “dysconnection” itself can be construed as having at least two somewhat distinct meanings. In one, the term represents the sense in which Blueler and Kraepelin thought of schizophrenia: a kind of “splitting of the mind” (32, 33). In all likelihood, this meaning carried only a vague relationship to neurobiological considerations. In his writing, Bleuler clearly related the idea to loss of cohesion in intellectual faculties, but the work of these great neurologists predated modern neuroscience, and thus they were not privy to the multitude of experimental methods and theoretical ideas available now. Indeed, in their time, understanding of brain function was largely grounded in phrenological/localizationist theories wherein the outputs of individual brain regions, emergent as their “functions,” were assumed to map onto anatomical structures in relatively direct ways (34, 35).

A second, more literal sense of dysconnection (or “disconnection” as originally proposed), is quite explicitly neurobiological: in this view, schizophrenia is an emergent behavioral phenotype resulting from profound alternations in the connectivity of the brain’s anatomic and functional pathways (18, 36, 37). Just as structural connectivity loosely constrains the brain’s functional architecture (38, 39), impaired anatomical connectivity most likely offers loose constraints on the inability of the brain in schizophrenia to integrate functional signals across regions in both non-task and task-active states (40, 41). It has been asserted that normal perception and cognition rely on a cortico-cortical phase synchronization mechanism that operates in conjunction with reentry to provide context for local cortical computations by way of inter–areal interactions (42). This mechanism normally provides a balance of integration and segregation to complex dynamic cortical networks, and schizophrenia is likely to be marked by a shift toward segregation such that local cortical areas express their information content without benefit of the context normally provided by interaction with other areas (43, 44). A modern view of the dysconnection syndrome constitutes a program for neurobiological discovery and offers the promise for arriving at fundamental insights on brain mechanisms that mediate the emergence of the illness.

## From Localization to Network Function: Neurology and Neuropsychiatry

The localizational model was in part driven by the technologies available for collecting data from the brain. In essence, the model relied on systematic analyses of functional loss in patients with localizable neurological lesions (45). Lesion-based models were characterized by rudimentary system’s-based approaches toward brain–behavior relationships, the Wernicke-Geschwind model of speech comprehension and production (46) serving as a good example. However, their accuracy was compromised by impoverished data and, more fundamentally, by the untenable (yet implicit) assumption that brain regions existed in fairly specific one-to-one relationships with overt behavior (34). Among others, Wernicke and Luria should be given credit for creating a hybrid model that distinguishes between elementary functions expressed by individual brain regions and complex functions that are properties of distributed systems of brain areas (42). Thus, as we understand it now, early neurology turned out to be an impoverished framework for understanding both the complexities of normal brain function and the complexities of disorders like schizophrenia.

The “neuron theory,” largely motivated by the work of Santiago Ramón y Cajal (47), emerged in the late nineteenth century. The theory experimentally developed the idea of neurons as functional units, the extracellular outputs of which reflect basic properties of behaviors (both simple and relatively complex). The neuron theory has been a (perhaps *the*) *sina qua non* of modern neuroscience. The explosion of single unit extracellular recordings has provided a wealth of insight into the complex response properties of single units and the extent of (particularly sensorimotor) function that these responses explain (48, 49). Nonetheless, theories of structure–function relationships based on single unit recordings are ultimately subject to the same ontological limitations as phrenology (50). Cognition and behavior (normal or pathological) are far too complex to reduce to single brain regions, let alone units. Indeed, as connectivity is a basic property of neurons (which connect through axons and synapses), connectivity studies are a natural direction for neuroscience. If anything, neurological models, neuron theories, and lesion studies provide evidence of only one of the multiple organizing principles of brain function at the macroscopic scale: the principle of relative specialization of function. This principle suggests that brain regions are more likely to sub serve one class of functions than another (51, 52). Relative specialization is a question of degree. There is little about the brain that is strictly “categorical.” Rather, it is more likely that the degree of specialization is relatively strong in sensorimotor or modality specific regions (“unimodal” regions), but relatively weak in regions involved in sensorimotor integration and “higher” behaviors [or “heteromodal” regions (53, 54)]. Clearly then, a different and parallel principle of functional brain organization is needed that more directly speaks to the emergence of complexity, and by corollary, the emergence of complex disorders of the brain.

If we treat the single neuron theory as the first revolution in modern neuroscience, the application of complex systems theories, and the operationalization of these analytic frameworks for understanding the brain must constitute the second (55). Ludwig von Bertalanffy’s work on general systems theory continues to reverberate in modern neuroscience (56) as the drive to explain processes in terms of interactions between the components of a system gains force (57). This is particularly pressing in terms of understanding how cognitive ontologies arise from the brain (39, 58). The functional integration of signals across brain regions presents itself as a parallel organizing principle of brain function (52, 59), and if complex cognitive ontologies arise from brain network interactions, and schizophrenia itself is in part defined as a “cognitive” illness (10), then it is reasonable to assert that the disorder results from impaired functional integration of signals across brain regions, i.e., brain network dysfunction.

The history of neuroscience is characterized by a synergistic relationship between methods and theory (60, 61). Theoretical advances in the neurobiology of schizophrenia have been crucially driven by developments in *in vivo* whole brain functional neuroimaging, in particular fMRI. fMRI is based on complex hemodynamic spatiotemporal signals (62, 63) that themselves lie at the apex of a series of complex neuronal (and presumably neurochemical) processes; these processes exist in uncertain relationships with the overt fMRI signal (22, 61). Thus uncovering brain network function and dysfunction from fMRI time series data has been termed a process of “reverse engineering” and “network discovery”; what must be engineered or discovered are the hidden states of brain function that give rise to fMRI signals (55, 64). If understanding brain network interactions is a process of quantitative discovery, then understanding of what the inferred processes are must be grounded in quantitative models applied to fMRI time series data (65).

## Models for Inferring Network Function: What Can be Inferred?

Distinct classes of analytical techniques have been fruitfully applied to fMRI time series data (38, 66, 67), and much of what follows is a synthesis of published and influential reviews. Our goal is to selectively sample and distil for the reader quantitative bases of analytic methods, and reveal what they render as knowable about brain connectivity and, by extension, dysconnectivity. Ultimately, connectivity is an inference that is based in the quantitative models used to assess it. This inference inherits the advantages and limitations of the models applied to discover these patterns in overt fMRI signals. In understanding brain network dysfunction in schizophrenia (or any psychiatric condition) exposure to the fundamentals of the analytic methods is a necessity for understanding what is being modeled (and ultimately inferred).

Functional connectivity (FC) and effective connectivity (EC) are two broad classes of analytic methods for assessing connectivity. FC generally refers to the statistical relationship (in the time domain) of two spatially distinct signals. In fMRI data, these analyses typically constitute calculations of bivariate temporal correlations usually at zero-lag thus ignoring potentially useful information about timing relations between BOLD time series drawn from distinct regions of interest, wherein strongly correlated or anti-correlated regions are “functionally connected” (68). In general, co-variations in time series signals are heavily used in assessing functional relations between elements of complex systems (69) and provide a useful though limited framework for network discovery. In the context of fMRI data, these methods have been noted for some limitations. First, they are sensitive to the variability of hemodynamic response functions (HRFs) across brain regions. Second, due to the limited temporal information in the signal, they generally must rely on “zero-lag” analyses, thus ignoring potentially useful information about timing relationships between BOLD time series. Moreover, they lack functional transitivity, in that the techniques are insensitive to divining functional relationships between regions that are correlated with a mediating time series (55, 70, 71). Coherence is a complementary measure of FC that estimates linear time–invariant relationships between multiple time series even at phase delays. As a spectral analog of bivariate correlational analyses, coherence accounts for phase relations in the cross-correlational function (while limited by the bandwidth of the hemodynamic response) (72). Finally, we also note the value of Wiener–Granger Causality (73), a method for inferring functional relationships between regions based on temporal predictability between time series. Granger Causality has also been referred to as directed FC (dFC), distinguishing it from undirected FC (uFC) methods that depend on correlative statistics (74, 75).

The standard notion of EC is that it captures the effect that one neuronal population exerts over another (regardless of the scale at which these interactions are assessed) (52). More specifically, true EC depends on capturing the relatively precise timing relationships between *neuronal* populations (76), thus depending on the applied models to capture the temporal dynamics of neuronal populations. Implicit within the definition of EC is that these techniques explicitly seek to model “causative” relations between brain regions and that they depend on generative network architectures of the brain. A causal relationship is both an ontologically different claim than simple statistical covariation, but importantly therefore also includes information about the direction of influences between regions (77). Moreover, EC entails a notion of estimated “coupling”: that is, the determination that a causal influence exists between neuronal populations fundamentally relies on constructing a *generative model* of that coupling. This means that understanding EC is a question of model comparison: what model of the brain is most likely to have generated the observed data, where each model itself constitutes a hypothesis of brain function in that context (78). The implicit and explicit assumptions of each approach and the models implemented therein exert constraints in inferences regarding brain network connectivity, and by extension, about dysconnectivity in schizophrenia. Beyond this, modeling can help unravel the physiological mechanisms underlying causal influences in the brain. That is, what is the effect exerted by the neurons in one brain area on those in another area. It is plausible that inter-regional interactions are modulatory, shaping the activity generated by the internal dynamics of the local area (42). To evaluate this hypothesis, we need to be informed by (a) the location on the recipient neurons of the synapses coming from transmitting neurons in other areas and (b) the neurophysiological effect of inter-regional influence (e.g., how does it change the sub threshold membrane potential?). Moreover, model *structure* plays an important inferential role. The likely generative model for schizophrenia may be different than that for controls, and the differences in model structure may provide valuable information regarding the architecture of the disease (41). These issues are revisited later.

## Undirected Functional Connectivity Techniques Based on Bivariate Correlational Approaches

Bivariate correlational approaches toward fMRI time series data are by far the most commonly applied measure of assessing temporal relationships between regions of interest. These methods make weak assumptions regarding functional transactions between regions, are frequently used in an exploratory manner, and do not provide measures of coupling in the manner of other techniques (see more on this aspect below) (75). Generally considered, this class of FC analysis mines statistical dependencies in the time domain (for fMRI data) between disparate time series. This approach is represented in:

$$\rho_{x,y}=\frac{\text{cov}\left(x,y\right)}{\sigma_{x}\sigma_{y}}$$

In this generic form, the correlation coefficient, ρ*_x,y_*, of two independent time series *x* and *y*, is equal to the covariance of *x* and *y* normalized by the product of the SDs, σ, of both signals. FC is an *emergent* statistical property of inter-relationships between time series, such as provided by the fMRI BOLD signal. Crucially, FC analyses have not usually relied on biophysical models linking neuronal with hemodynamic responses (79), which means the notion of “coupling” in uFC analyses does not extend beyond the statistical realm. Thus, as with any emergent statistical result, the only “model” of function tested by uFC methods is the null model, that is, testing against an absence of significant correlation between brain regions. As a result, these methods make few assumptions about temporal context, the temporal scale, or resolution of any putative underlying process.

The weak assumptions relating to processes, however, also confer some advantages for this class of FC measures. There is greater statistical reliability with shorter time series than measures that make stronger assumptions or that require more precisely modeled time series (as will be seen with Granger Causality techniques). Moreover, weak assumptions also mean that uFC analyses are relatively robust to contributions of filtering or slice-time correction applied to fMRI data that can artificially disrupt the fine-scale temporal structure of the signal, thus producing spurious causality (we discuss the relationship between temporal information and causal inference in detail below).

## uFC Techniques Based on Coherence

Coherence is a complementary approach to time-domain uFC that has enjoyed widespread use with EEG data, but has been generally under-utilized for fMRI analysis, largely because limited temporal information in the fMRI signal preempts complex spectral analyses of fMRI signals. Coherence is a frequency-domain measure of how well one signal linearly predicts a second in a time–invariant fashion. The most common approach defines coherence as the magnitude of the cross-spectrum of two signals, *x* and *y*, normalized to the power spectra of each signal. Specifically, magnitude squared coherence, *Coh_xy_*, at a given frequency, *f*, can be defined as

$$Coh_{xy}\left(f\right)=\frac{{\left|P_{xy}\left(f\right)\right|}^{2}}{P_{xx}\left(f\right)P_{yy}\left(f\right)}$$

where *P_xy_* is the cross-spectrum, and *P_xx_* and *P_yy_* are the power spectra of both signals. As with correlation, the metric is scaled ($0\leq Coh_{xy}(f)\leq 1$), such that 0 represents no linear relationship between the two signals, and 1 represents the ability to perfectly predict one signal from the other. In order to reduce the variance and edge artifacts that can be introduced by windowing data in the time domain prior to a Fourier transform, coherence is often calculated using Welch’s modified periodogram averaging method (80). An estimation of the interregional coherence can then be calculated for each resulting frequency bin or averaged over the frequency range inhabited by the hemodynamic response function (HRF), typically defined as 0–0.15 Hz (70).

The time–invariant property of coherence allows the measure to assess relationships between time series beyond the zero-lag constraint. As previously noted, variations in the shape of the HRF along with time delays may offset the temporal progression of two related BOLD responses in functionally related brain regions. This property of fMRI signals presents a challenge for the sensitivity of correlation analyses, because correlations will decrease toward 0 as a function of increasing lag between two otherwise synchronized signals. For example, if two brain regions are co-modulated by a task, but with a time delay or with different hemodynamic responses, they may be synchronized with a phase lag. This can be generalized as the relationship between a sine function, sin(t), and a second identical, but phase-shifted sine function, sin(t+θ). As the phase shift, θ, progresses from 0 to $\frac{\pi }{2}$, the zero-lag correlation decreases to 0, then further to −1 as θ approaches π. However, a linear relationship still exists between the two signals, and coherence between the two sine waves will remain at 1. In other words, coherence allows for phase shifts or temporal lags when scoring the FC. Thus, while both zero-lag correlation and coherence are able to capture the relationship between the signals when the phase lag is near 0 or π, lags around $\frac{\pi }{2}$ are lost to zero-lag correlation [for more details on this method, see Ref. (71, 80)].

Spurious non-zero values of coherence can arise in the analysis of physiological time-series data simply due to the spectral properties of the signals (81). An effective method for correcting this bias is the use of surrogate data sets (81–83). Here, the time series data are shuffled, thus disrupting the phase relationships but preserving the statistical distribution of the spectral content. After multiple iterations, the mean and SD of the surrogate results can be compared to the experimental results. Any values of coherence exceeding the 95th percentile of the surrogate data distribution can be considered significant.

Correlative analyses are undirected by definition – hence the label “undirected FC” above. An interpretational limitation is that these methods are agnostic regarding directional influences between network nodes. Undirected connectivity/coherence analyses have a different footprint in the analyses of electrophysiological signals where within- and inter-regional coherence can be assessed at multiple frequencies (84, 85), each of which reflect somewhat distinct functional properties of cortical function. The temporal resolution of fMRI data does not afford this luxury. Nonetheless, there has been an exuberant profusion of undirected FC techniques applied to the resting state fMRI. Combined with graph-theoretic methods, these applications have provided insight on network disorganization in schizophrenia (40, 44). A valuable extension of this work would be in understanding how these altered network hierarchies in schizophrenia are expressed in disconnection in a task-active state. However, such extension would require integrating multiple areas of focus, including within-subjects acquisition of resting *and* task-based data, and the implementation of multiple techniques for estimating connectivity (outlined herein). Nevertheless, we suspect that the value of uFC analyses alone in inferring disconnection in schizophrenia is limited. The applied statistical model for uFC is relatively impoverished and identifies emergent statistical properties of fMRI signals [see Ref. (16) for a more comprehensive treatment of these questions]. These emergent statistical properties are removed from biophysical models linking accumulative neuronal with hemodynamic responses (86) and therefore may be distant from mechanisms of brain function.

Understanding directional relations between network constituents is important for multiple reasons. While various brain areas have reciprocal structural connections, it is highly unlikely that directional relations will simply reflect structural connections. Moreover, for a variety of reasons, structural connectivity only offers lose constraints on the functional integration of signals. In terms of functional organization, it is very likely that the direction of information flow in the brain is of critical importance for organizing cognitive functions and consciousness. This has been demonstrated from numerous studies of connectivity in both EEG and fMRI (87, 88). Moreover, directional effects are also important in the context of brain network hierarchies (53, 54). Control regions of the brain, including the dorsal prefrontal cortex and the anterior cingulate cortex enjoy higher hierarchical status within the overall system (74, 89, 90), suggesting that their functional transactions are likely to be asymmetric (91). Capturing these asymmetries will prove highly valuable, particularly in schizophrenia, which has frequently been characterized as a disorder of cognitive control (92–94). These considerations motivate quantitative FC methods that explicitly attempt to capture directional interactions between network constituents in health and schizophrenia. We next consider two directed FC methods, Granger Causality (73) and psychophysiological interaction (PPI) (95, 96).

## FC Techniques Based on Directional Approaches

### Psychophysiological Interaction

Since its introduction in 1997, PPI has constituted a widely used approach to directed FC (95). PPIs are constructed by extracting a time series from a seed region of interest and multiplying its activity with a stimulus function or regressor encoding the psychological context (95). This computation generates a regressor term that is used to capture variance in the time series of target voxels, as explained by the seed region, within the context of the task. Technically, signals that are highly predictable will produce a significant PPI effect but PPIs are readily distinguished from correlative methods. This follows because they test for second order dependencies. In other words, they test for a linear dependency of activity in the target region on activity in the seed or source region that itself depends upon another (psychological) variable. It is this high order, or interaction, effect that breaks the symmetry and endows PPI analyses with a directed nature. Strictly speaking, one could argue that PPIs reflect a simple (GLM) model of EC. However, we associate a PPI analysis with the inference that there are statistically significant second order dependencies; namely the interaction. As such, we will treat PPI analyses as a form of directed FC (i.e., statistical dependence).

The GLM approach to assessing PPIs provides a potentially more nuanced framework for modeling the time series data as it allows the model to co-vary out confounds. This is accomplished *via* a point-wise multiplication of the seed time series with a stimulus function and the inclusion of various sources of noise in the model (96). This product time series is then the interaction between the BOLD time series and the psychological task – the eponymous PPI. The interaction time series, along with both the task model and HRF time series, can then be used as regressors in the GLM, which separates the variance in the target signal associated with the psychological task, the HRF, and the interaction between the two signals (the psychological regressor and the response in the seed region). The equation below captures the directional bases of PPIs:

$$y_{i}=ay_{0}+b\left(y_{0}\times u\right)+cu+X\beta$$

The above is readily distinguishable from the typical GLM applied in activation models by the presence of the asymmetric interaction term $\left(y_{0}\times u\right),$in which regressing $\left(y_{0}\times u\right)$ on $y_{i}$ is asymmetric with regressing $\left(y_{i}\times u\right)$ on $y_{0}$ (55).

As with all modeling of fMRI signals, PPIs constitute an *a priori* conceptual model of brain function. The implicit model is that *contextual interactions* between seeds and targets can be characterized within a statistical framework. In this context, the choice of seed and the psychological context are free parameters of the model, and these choices must be well motivated by prior knowledge regarding task characteristics and the putative network profiles of the seed in an integrative network (94, 95, 97, 98). The simplicity of the PPI framework is advantageous as it affords rapid exploration of network profiles in normal and clinical populations (and differences between them). Nevertheless, this simplicity is also a limitation because comprehensive network interactions are rarely subsumed by pairwise interregional interactions. Moreover, PPIs are not defined by biophysical models linking neuronal with hemodynamic responses, and therefore they do not provide measures of neuronal coupling, limiting their neurobiological interpretation (95, 96).

### Granger Causality

In treating the brain as a complex system, computational neuroscience has successfully coopted analytic tools that were initiated for other disciplines, where many data properties are shared with fMRI data – particularly the idea that functional aspects of the system interactions are “hidden” in time series data. Notable examples from physics and electrical engineering include time-frequency analyses, graph theoretical approaches, and information theoretic measures. GC is a measure of directed FC, which has its roots in the analysis of economic data (99). Since its import into the field of neuroscience, GC has been used extensively in estimating directed connectivity relationships in multiple modalities of brain imaging including EEG, MEG, and fMRI (73). GC can, in brief, by described by:

$$X_{t}=\sum_{j=1}^{m}\alpha_{2j}X_{t-j}+\sum_{j=1}^{m}\beta_{2j}Y_{t-j}+\varepsilon_{2t}$$

$$X_{t}=\sum_{j=1}^{m}\alpha_{1j}X_{t-j}+\varepsilon_{1t}$$

$$GC_{Y\to X}=ln\left(\frac{\Sigma_{1}}{\Sigma_{2}}\right)$$

where, $\Sigma_{1}=\text{Var(}\varepsilon_{1\text{t}}\text{)}$ and $\Sigma_{2}=\text{Var(}\varepsilon_{2\text{t}}\text{)}.$ In GC, the estimated dFC depends on the model order and the estimated time lag between modeled time points in the two signals. Accordingly, appropriate specification of the time lag between different observations and the order or number of past observations included in the auto-regression model above are crucial for properly testing for GC influences. Generally, the appropriate time lags and model orders are unknown (although they can be estimated using procedures based upon mutual information and Bayesian model comparison). This means the ability of GC to make inferences about FC – based on relatively slow dynamics – is potentially challenging [see Ref. (100, 101)]. It should be noted that correlation also suffers from a dependence on time lag, and important correlations may be missed by over-reliance on the lag-zero value.

In general, GC evaluations may cover a range of time lags up to a maximum value in order to search the parameter space for the correct lag. This approach has been demonstrated for transfer entropy, an information theoretic extension of GC. Lee et al. (87) define the information flow between two nodes as the maximum normalized transfer entropy found by scanning the time delay parameter space. This method has been successfully applied to electroencephalograph data in humans (87), as well as electrocorticograph data in rodents (102), allowing researchers to track changes in cortical information flow across changes in states of consciousness. However, given the limited temporal resolution of fMRI, the model order rarely extends beyond one time step.

Because GC relies on the temporal progression of time series to estimate causality, it is crucial that the ordering of the time series data remain unmodified. The convolution process used in finite- and infinite-response filters alters each data point based on both the previous data points and those succeeding it. After filtering, then, the value at any given time has been influenced not only by its past but also by its future, thus undermining the basic assumption of causal inferences. Convolution with a HRF can also affect the structure of time series, notable because as referred to earlier, HRFs can differ across brain regions. Several lines of work somewhat mitigate against these concerns. There is evidence that GC is invariant to HRF convolution (103), and as noted earlier, assessing directional asymmetries (i.e., direction is used as a condition of interest in the GLM) of GC coefficients between regional pairs can better constrain the interpretation of causal effects (104).

Notwithstanding the directed nature of the applied models, as with PPI, GC is limited by a lack of adequate bases in physiological underpinnings. The dynamics of interacting neural populations is considerably removed from the signals recorded by fMRI and subsequently entered into a GC calculation, and there is no extant physiological model to bridge it. Nevertheless with appropriately constrained research questions and experimental designs, GC (as with PPIs) provides a quantitative characterization of directional pairwise interactions between constituents of brain networks. These insights can provide meaningful perspectives on the nature of dysconnection in schizophrenia.

## Effective Connectivity Techniques

The term “effective” connectivity is the source of much confusion, but can be more clearly understood from the perspective of interactions between constituents of a complex system. From a mechanistic perspective, EC has been defined as the influence that one neural system exerts over another (76). EC models thus attempt to embody dynamic and timing relationships between system constituents, typically using elements of control theory (52). Because there is no reasonable sense of a “true” and veridical model of brain function that can be wholly derived from observed data (15), EC relies on a method for evaluating *competing* model architectures for a set of network nodes, any of which is a plausible generator of the observed data, but have inherently differing likelihoods of having done so (78). This competitive (and ultimately Bayesian) framework is essential for the process of model discovery and is largely absent from previously considered techniques of directed and undirected FC (though some elements are present in the evaluation of directional asymmetries using GC). These motivations for divining neuronally plausible network-based mechanisms are by themselves insufficient unless a mapping from neuronal responses to the generated hemodynamic response is implemented (86). Dynamic Causal Modeling (DCM) incorporates a Bayesian framework for network discovery while using biophysical models relating neuronal to hemodynamic responses.

### Dynamic Causal Modeling

Dynamic causal modeling was introduced as a seminal framework for discovering mechanistic brain network function from fMRI (and other) data (105) and has subsequently received significant methodological inspection of its biophysical and probabilistic bases, and its reliability (106, 107). In its original conception, DCM represented the brain as a bilinear system (a lower order approximation for non-linearity) in which the inputs are the experimental conditions and the outputs are the hemodynamic response measured using fMRI. Because DCM is explicitly interested in modeling dynamics and changes in these dynamics in response to inputs, elements of control theory are incorporated where changes in network states are modeled using the following state differential equation:

$$\frac{dx}{dt}=\left(A+\sum_{j=1}^{m}u_{j}B^{\left(j\right)}\right)x+Cu$$

To be clear, this equation describes the dynamics of “network states” rather than of any physiological metrics. In other words, this is not an explicit physiological model. The state equation represents three differentiable components with large distinct bio-psychological extensions: here, *A* represents the matrix of endogenous coupling between brain regions. Simply speaking, this can be construed of as the hypothesized functional connectome underlying the evaluated model. The connections within any model represent a hypothesis on the pattern of connectivity. How the model is “wired” depends on a combination of priors that include reliably known connective pathways and/or explicit hypotheses for discovering which pathways may or may not exist independent of task-induced experimental changes. The variable *B*^(^*^j^*^)^ represents the modulatory response in the network connections due to changes in experimental conditions *u_j_*. Finally, *C* represents the direct driving input on particular regions as induced by the experimental conditions.

Network discovery with DCM relies on the identification of generative network architectures with the highest evidence given the observed fMRI data, thus testing hypotheses on an *a priori* defined model space. Thus, the space comprises neurobiologically plausible competing models, each representing hypotheses on the connective-architecture of the investigated neural system (78, 108, 109). Therefore, rather than using traditional goodness-of-fit metrics to assess the viability of an individual model, DCM relies on evaluating these multiple neurobiologically plausible competing network models, wherein across competing models, specific network connections may be permuted, and these permuted connections specifically serve as hypotheses. Thus, across models, connections can be constrained or informed by known properties of neuroanatomy (110) or may be permuted with some agnosticism regarding the specifics of the underlying anatomy itself. More importantly, the method provides a plausible approach toward understanding how brain networks “work,” by incorporating principles of both relative specialization and functional integration. By corollary EC methods provide a compelling context for understanding cases (such as schizophrenia) in which the brain does “not work” (111).

## Challenges for Inferring Connection and Dysconnection

Understanding *causal* antecedents of disease have historically driven the promise of successful medical intervention or pre-emption. This concept of *deterministic* causality is a fundamental assumption in medicinal discovery and practice. Illnesses have causes that can be determined and addressed. These causes may cut across biological, environmental, and epidemiological levels (112), yet it is commonly assumed that the causative pathway is, in fact, deterministic. Moreover, many medical successes have benefit from the relative simplicity of the structure–function relationships within the organs of relevance. For example, advances in cardiac or pulmonary treatment benefit from the relatively straightforward relationships between the structures of the heart or the lung, and their expression in function. Unfortunately, the brain proffers no simplicity in this regard. Rather, its structure (to the extent that it is fully knowable) provides relatively light constraints on its emergent functional interactions, and even more indeterminacy with respect to how overt behavior arises. This “degeneracy” is a significant challenge to assessing the brain’s structure–function relationships (35) and degenerate structure–function mappings (i.e., many to one mappings) can also be observed in the relationship between functional and effective connectivity. In other words, there are many connectivity architectures of EC that can produce the same FC. We will consider an example based upon a common source below. Technically, this means that inferring EC from FC can be an ill-posed problem that necessarily calls for prior constraints and (abductive) inference, as we have carefully intimated in the Introduction.

For instance, should connectivity between two regions be established *via* a statistical model, the conclusion that the two regions have a direct relationship can always be undermined by the hypothetical existence of a third, undetected member of the system, though this possibility can be somewhat mitigated by knowledge of the anatomical connectivity structure of the areas under consideration (thus, region C may only be a candidate driver if it is anatomically known to project to regions A and B). Thus, a bivariate correlation between regions A and B, may be driven by (1) a functional or causal relationship in which A causes B and/or B causes A, or (2) regions A and B are both modulated by a third region, C, such that A and B are synchronized without directly interacting. If there is a different time delay between C and A than between C and B, this can even appear as a phase–lag relationship between regions A and B thus presenting as a directed FC relationship. However, given that methods such as GC are based on predictability, they can be used to establish a driving role for area C (113). Thus, demonstrating a directed relation based on any measure of predictability is superior to phase-lag measures in terms of what can be inferred. Nevertheless, although multivariate GC can account for a third time series and its influence on the system, this can only be modeled when the third time series has been identified and is measurable. If the third node in the system is unknown, then contribution toward the inference of connection remains unknown. These limitations are a particular example of the general problem of causal inferences in brain networks, and as has been forcefully argued and extensively discussed (114, 115), deriving *deterministic causal inferences* regarding brain network interactions may be a fundamentally untenable exercise. Not only might the etiology of schizophrenia remain obscure, but even the inference of brain network mechanisms that might inform the proximate causes of the illness may suffer from fundamental challenges. The potential contribution of hidden or latent nodes is, in principle, not a problem for models of EC like DCM. This is because one can use Bayesian model comparison to evaluate the probability of a hidden common source – by comparing models with and without hidden nodes.

## What Aspect of Dysconnection in Schizophrenia Can be Understood from the Analyses of fMRI Signals?

**Figure 1:**
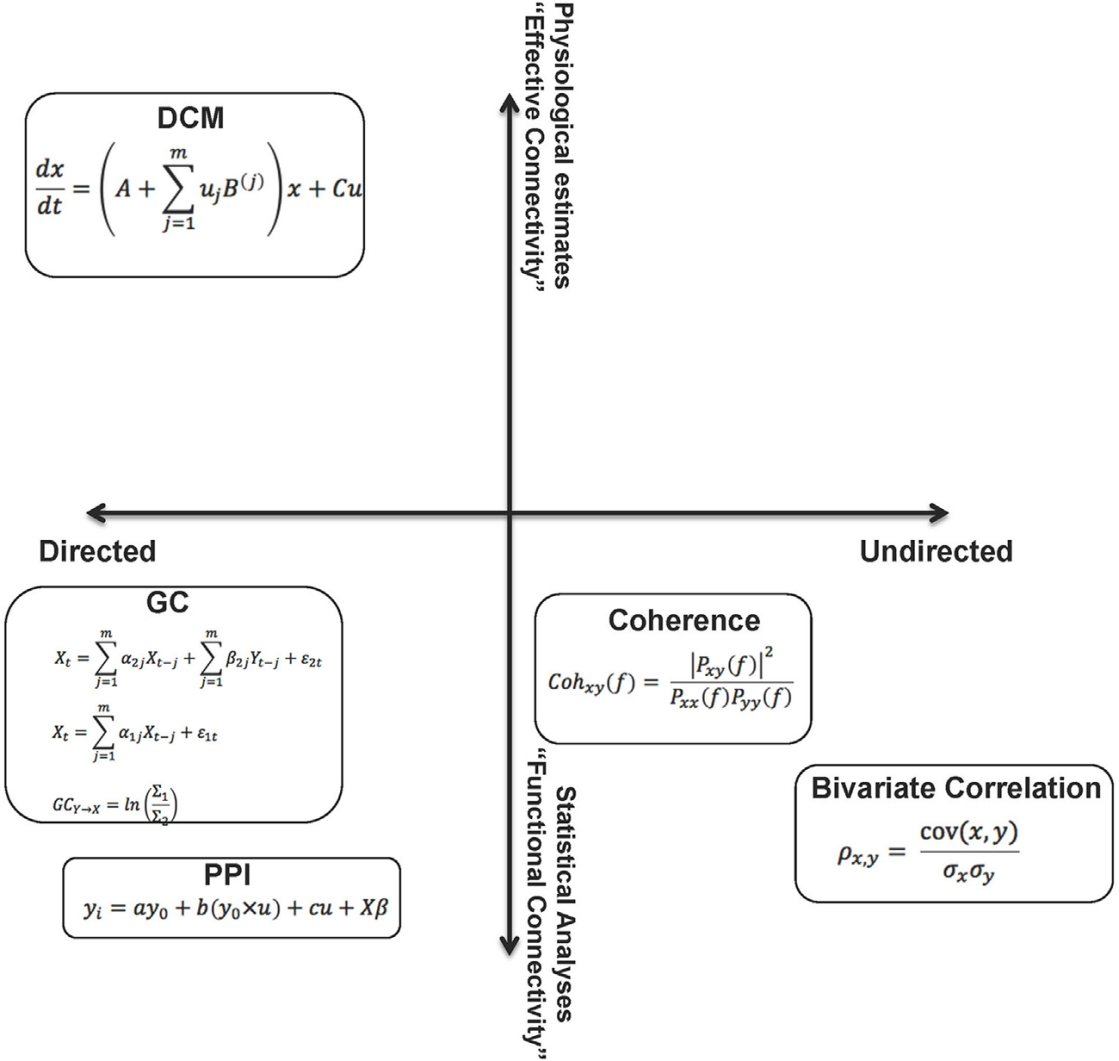
**The figure summarizes the methods visited in this overview, by placing them in a two dimensional “space.”** From this perspective, the relative (to each other) properties of these analytic methods are somewhat apparent on each dimension. These dimensions can be associated with the “strength” of each method. A reasonable characterization of “strength” can be the degree to which the parameters of the connectivity models can be linked to biophysical processes or computation in real neuronal networks. The constraints on parameters emerge from assumptions within the models (functional to effective connectivity) and also relate to the notion of directionality within models (directed to undirected). As noted in the text, though the space cannot be defined by metric properties, some aspects of ordinal relationships between the methods can be inferred.

Figure 1 summarizes the methods visited in this overview, arranged in a two-dimensional conceptual space. The space carves out quadrants within which the methods are defined by their directionality (directed vs. undirected network interactions) and the relative “strength” of the methods (functional or effective connectivity), where “strength” can reasonably be construed of as the degree to which the parameters of connectivity models can be linked to biophysical processes or computation in real neuronal networks.

This is a parsimonious representation, yet provides a panoramic (and somewhat self-evident) overview of the different aspects of network dysfunction that can be inferred from each class of analytic approaches applied to fMRI time series data. Though we emphasize that the axes do not approach metric properties, we use weak ordinality to arrange the methods within the space to allow some contemplation of their strengths and weaknesses. For example, directed analyses may provide more interpretational value than undirected analyses, and EC approaches may, in principle, be more desirable than FC approaches. A crucial dimension not represented is tractability of implementation – both in terms of designing analyses and computationally implementing them. We note that this tractability is unevenly distributed within this space, yet is an issue of concern in the search for inferring dysfunction in schizophrenia. We also note that the motivation for this review relates to schizophrenia, yet the extensions are general across psychiatric illness (though the networks of focus within the brain may be idiosyncratic to the phenotypes of interest).

Is schizophrenia itself *tractable*? It is unclear whether all aspects of the etiology of this complex condition are knowable, yet inferring dysconnection in schizophrenia is a special case of inferring brain network function. In that sense, inferring the dysconnection syndrome is perhaps no more or no less tractable than understanding how macroscopic brain network interactions can be related to other overt or covert behaviors. We suggest that if fMRI has told us anything of significant value, it is that macroscopic brain network dynamics expressed at the scale of seconds can be successfully modeled to infer aspects of brain function. Theory and technique now offer avenues for inference and discovery that did not exist even in the recent past. The methods covered herein (and others) offer the prospect of inference and discovery that suggests the promise of significant mechanistic understanding of schizophrenia.

## Author Contributions

VD directed the writing of the manuscript and contributed the principle conceptual direction. SB provided important conceptual insight and technical language particularly relating to Granger Causality models. BS provided insights relating particularly to functional connectivity methods.

## Conflict of Interest Statement

The authors declare that the research was conducted in the absence of any commercial or financial relationships that could be construed as a potential conflict of interest.
